# Morphological analysis-based yield modeling in greenhouse grown cherry tomato (*Solanum lycopersicum*) under prolonged heat stress

**DOI:** 10.3389/fpls.2025.1730694

**Published:** 2025-12-19

**Authors:** Sumin Kim, Jaehak Jeong, Sojung Kim

**Affiliations:** 1 Department of Environmental Horticulture and Landscape Architecture, College of Life Science and Biotechnology, Dankook University, Cheonan-si, Republic of Korea; 2 Texas A&M AgriLife Research, Texas A&M University, Temple, TX, United States; 3 Industrial and Systems Engineering, Dongguk University-Seoul, Seoul, Republic of Korea

**Keywords:** APEX, cherry tomato, heat stress, priming, greenhouse climate model

## Abstract

In South Korea, cherry tomato (*Solanum lycioersucum*) is a major greenhouse vegetable crop. However, climate change has steadily raised Earth’s average temperature, posing a serious challenge for greenhouse agriculture. Elevated temperatures can trigger heat stress in greenhouse crops, leading to considerable yield losses. This study developed a greenhouse tomato growth model for two cherry tomato accessions, HR17 and HR24, cultivated under heat stress conditions during growing periods. Climate projections based on polynomial regression were incorporated into the plant growth model to assess climate change impacts on tomato yields. The two tomato accessions demonstrate distinct growth characteristics: HR24 allocates more biomass relative to fruit yield (Harvest index:0.48), whereas HR17 shows greater fruit production than biomass accumulation (Harvest index:0.65). Their yield responses also vary under future climate scenarios highlighted by temperature increases of 1-8°C and extended hot seasons compared to historical records. HR24 appears more resilient to heat stress than HR17. Under Climate Change scenarios (SSP245 and SSP585 pathways), HR17 will decrease its fruit yield by around 1.2 Dry Ma/ha, while HR24 yields will be increased by round 1.3 Dry Mg/ha. This increased tolerance in HR24 may be attributable to its ability to sustain photosynthetic activity through higher production of biomass organs such as leaves and stems. These findings form a foundation for developing greenhouse crop models in future research and supporting farmers by providing more reliable yield forecasts.

## Graphical Abstract

**Figure:**
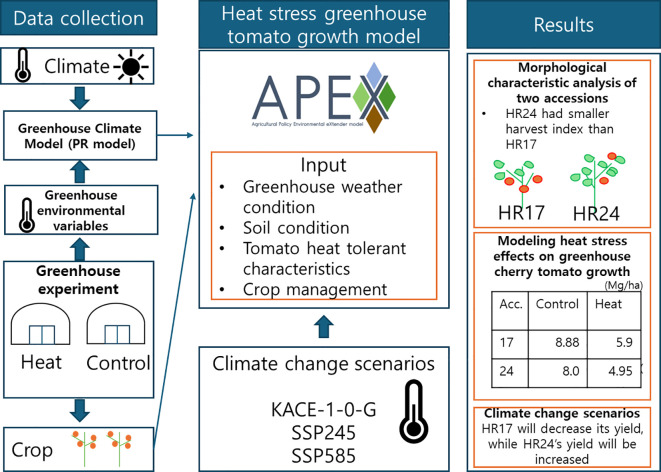
Schematic of modeling effects of pro-longed heat stress on greenhouse cherry tomato yields. The greenhouse cherry tomato models used weather data generated by Greenhouse Climate model, and the crop parameters were developed based on the 2-years of greenhouse experiments.

## 1 Introduction

Cherry tomato (*Solanum lycopersicum*) is a widely cultivated horticultural crop in South Korea, recognized for its high economic value, nutritional content, and flavor, and its production continues to increase nationwide. The annual output of cherry tomatoes was approximately 355,107 tons in 2017, rising to 369,383 tons by 2021 (Statistics Korea, 2025). The optimal temperature for tomato growth ranges between 25°C and 27°C, with a base temperature of 5°C (Statistics Korea, 2025). Although tomato plants adapt to various climate conditions, they are notably susceptible to heat stress. When temperatures exceed 30°C, respiration over-takes photosynthetic nutrient accumulation, resulting in stunted growth and increased incidence of flower drop. In addition, high temperature can cause male gametophyte abortion, decreasing fruit set (Alsamir et al., 2021). With intensifying and more frequent heat waves in summer attributed to climate change, heat extremes pose an increasing global threat to stable crop production.

Several studies have investigated the effects of heat stress on physiological, morphological, and biochemical changes in tomato plants (Alsamir et al., 2021; Siddiqui et al., 2017; Zhou et al., 2019). According to Xu et al (Xu et al., 2017), membrane damage is a primary symptom of heat injury, with heat stress positively correlated to the rate of electrolytic leakage. Heat stress disrupts cellular and metabolic processes, such as respiration and photosynthesis. It induces alterations in energetic capacity (e.g., ATP and ADP levels) in tomato leaves and affects the accumulation of Rubisco isoforms, ultimately reducing photosynthetic efficiency (Parrotta et al., 2020). Additionally, heat stress impairs tomato reproductive development. Extended periods of moderate heat result in decreased pollen production, lower pollen viability, reduced pollen shedding, diminished ovule viability and stigma receptivity, and greater physical separation between stigma and another cone (Kinet and Peet, 1997). In response to yield losses associated with heat stress from climate change, developing “heat-tolerant varieties” of tomato is essential, enabling farmers to sustain high productivity despite rising temperatures.

Determining the primary physiological and morphological characteristics contributing to heat resistance is essential to developing heat-tolerant varieties effectively. Following this, it is necessary to assess the performance of selected heat-resistant varieties under heat and other abiotic stress factors (e.g., drought). Employing models to evaluate the effects of heat stress on the growth of various tomato varieties provides an efficient approach for identifying heat-resistant lines capable of maintaining yield under stress. While numerous field and laboratory studies have assessed the influence of heat stress on physiological, morphological, and biochemical responses in tomato varieties, there is a lack of studies monitoring tomato growth under prolonged heat exposure (more than 7 days). The prolonged heat exposure is the condition when temperatures exceed critical thresholds for prolonged durations to disrupt physiological and biochemical processes in plants (Moore et al., 2021). Prolonged heat stress testing is critical to find individual recovery growth potential that may not be apparent in short-duration tests.

In this study, a tomato growth model was constructed, and tomato yields were assessed under increasing temperature scenarios. For model calibration and accuracy, a greenhouse experiment was performed to compare growth responses to heat stress in two varieties characterized by differing levels of heat resistance. This research constitutes the first simulation of tomato yield under continuous heat stress conditions. A greenhouse climate model has been established to project tomato yields in future climates to simulate temperature and humidity dynamics under anticipated climate change scenarios. The resulting simulations are intended to inform breeders and policymakers of potential long-term effects of heat stress on tomato yields across various temperature rise scenarios.

## 2 Materials and methods

### 2.1 Field design and morphological and yield measurements

**Figure 1:**
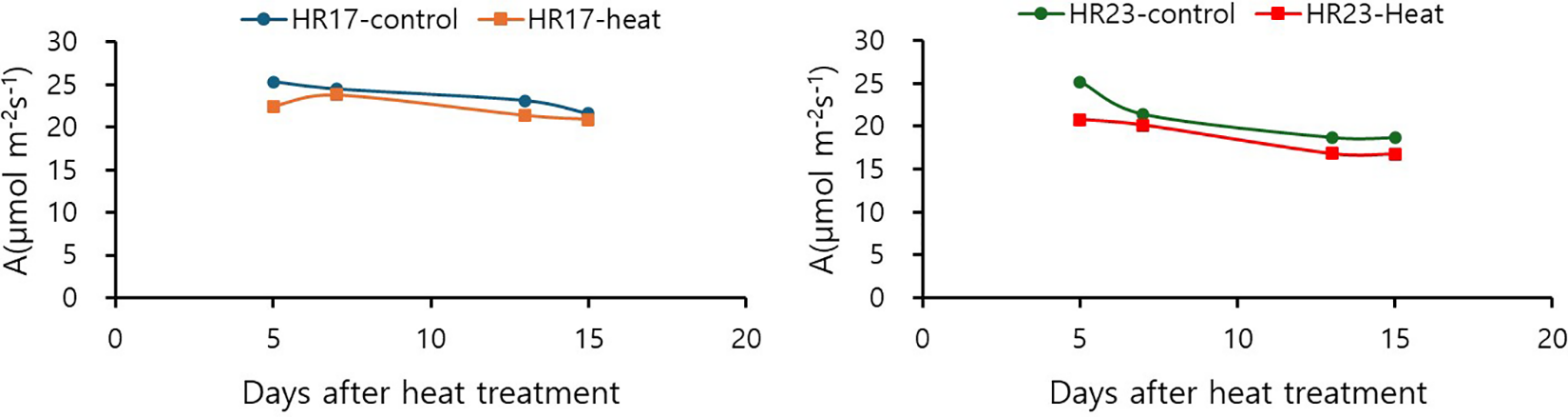
Average daily temperature fluctuations in heat-treated and control greenhouses during 2022 and 2023 (Wanju, Republic of Korea). The red circle indicates temperatures in the heat-treated greenhouse, and the blue triangle illustrates temperatures in the control greenhouse. The table presents the total number of treatment days and the number of days when daily maxi-mum temperatures exceeded 40°C in each greenhouse.

Two cherry tomato (*Solanum lycopersicum*) accessions, HR17(a moderate heat-tolerant, National Institute of Horticultural and Herbal Science, Wonju, Republic of Korea) and HR24 (a heat-tolerant commercial cultivar, Joeungyeo’, Farm Hannong, Seoul, Republic of Korea), were evaluated. HR17 produces round shaped fruit, while HR24 produces oval-shaped fruit. The field experiments were performed in two temperature-controlled greenhouses at the National Institute of Horticultural and Herbal Science (Wanju, Republic of Korea, 35°50’N, 127°03’E) during 2022 and 2023. Prior to heat treatment, 1.5-month-old seedlings of HR17 and HR24 were maintained under control conditions in the greenhouse for two weeks, after which the two greenhouses were set to different temperature regimes on May 18^th^. The control greenhouse was maintained at 30°C during the day, while the heat-treated greenhouse was maintained at 35°C during the day. The average air temperature in the control greenhouse ranged from 25 to 35°C, while the air temperature in the heat-treated greenhouse was consistently 2-5°C higher than the control greenhouse (**Figure 1**). During the nighttime, the average temperature in control was around 20°C, while the temperature in heat stress condition was around 21°C. Ventilation was used to control temperatures in greenhouses. The number of days with maximum daily temperatures exceeding 40°C in the heat-treated greenhouse was 28 days in 2022 and 29 days in 2023 (**Figure 1**). Relative humidity was maintained between 50% and 85% in both greenhouses. Plants received regular irrigation using a drip irrigation system and were fertigated weekly with nutrient solution A (N 5.5%, K 4.5%, Ca 4.5%, B 0.00014%, Fe 0.05%, Zn 0.0001%, and Mo 0.0002%) and nutrient solution B (N 6%, P 2%, K 4%, Mg 1%, B 0.05%, Mn 0.01%, Zn 0.005%, and Cu 0.0015%) (Mulpure, Daeyu, Seoul, Republic of Korea).

The experimental plot was arranged as a split-block design with replicates over two years. Temperature treatments (control and heat) served as the main plot, while the subplots were the two accessions, HR17 and HR24. Main plots corresponded to the separate greenhouses. Within each greenhouse, subplots were arranged in a randomized complete block design with three replicates each. In every block, five plants of each accession were planted at 30 cm spacing. The row-to-row distance was set at 140 cm.

Plant height and stem thickness were measured regularly during the treatment period. For the first week after heat treatment initiation, measurements were taken every two days, followed by measurements every two weeks until the final harvest. Plant height(cm) was measured from the base of the plant to the tip of the highest branch. Stem thickness(mm) was determined using a digital caliper (CD-20APX, Mitutoyo Co., Ltd., Kanagawa, Japan).

Due to the occurrence of plant diseases in 2022, harvest dates varied between years. In 2022, plants were harvested on the 34^th^ and 55^th^ days following heat treatment, whereas in 2023, harvests occurred on the 34^th^ and 77^th^ days after heat treatment. For both years, measurements at harvest included fresh total weight(g), total fruit weight(g), number of fruits per plant, leaf area index, height(cm), and stem thickness(mm). After harvest, plants were dried in a 65°C oven until a constant weight was achieved. The samples were heated in the oven until its mass no longer changed. Moisture content was determined by subtracting the dry weight from the fresh weight. The harvest index was computed using fruit weight in relation to total fresh weight. Leaf area per m2 covered by the plant was recorded using a LICOR-300 device (Lincoln, NE, USA).

On days 5, 7, 13, and 15 after heat treatment in 2023, net photosynthesis rate (A, µmol m-2s-1) was evaluated using a gas exchange system (LI-6800, LI-COR Inc., Lincoln, NE, USA). Measurements were conducted on a newly fully expanded leaf between 10:00 and 14:00. Instrument temperature settings were maintained at 25°C (non-stress temperature) for control and 35°C (moderate heat stress temperature) for heat treatments. Light intensity was set to 600 µmol m^-2^s^-1^, and CO_2_ concentration was maintained at 400 µmol m^-2^s^-1^ with 60% relative humidity in both greenhouse environments. Leaves were exposed to various irradiation levels for 4–5 minutes, after which data collection was performed. The photosynthesis rates collected under each treatment were used to determine radiation use efficiency (WA) within the plant parameter set.

The influence of heat treatment and accession was statistically assessed using analysis of variance (ANOVA). Year was considered a random effect, while heat treatment and accessions were regarded as fixed effects. All statistical analyses were conducted using the SAS software package (SAS 9.4, Cary, NC, USA).

### 2.2 Development of a greenhouse climate model

For the development of the climate model, data on maximum and minimum temperatures (T_max, T_min) and humidity (RH) in a greenhouse were collected during the experimental periods in 2022 and 2023. In addition, external weather data were obtained from the Agri-cultural Weather 365 website, which is available at http://weather.rda.go.kr (accessed on 1^st^ February 2023). **Table 1** provides a summary of monthly weather conditions during the experiment periods. A polynomial regression (PR) model is employed to characterize the nonlinear relationship between relative humidity and climate variables. The PR model is advantageous as it is mathematically practical, being expressed as a formula similar to standard regression models, and, in contrast to deep learning methods, allows analytical modeling with relatively small data sets (Kim et al., 2022). (Equations 1, 2) describe the general structure of the PR model.

**Table 1:** Weather conditions outside the greenhouse in 2022-2023.

Year	Month	Solar radiation (kWh/m^2^)	Relative humidity	Maximum temperature (°C)	Minimum temperature (°C)	Precipitation (mm)	Wind speed (m/s)
2022	May	23.76	0.59	27.36	15.12	0.52	1.68
	June	17.68	0.73	28.82	19.79	5.47	1.63
	April	16.46	0.78	31.11	23.72	5.94	1.56
	August	18.13	0.79	33.04	26.01	1.39	1.66
2023	May	16.05	0.71	25.51	16.64	11.30	1.40
	June	19.95	0.70	28.86	19.18	5.66	1.56
	April	14.59	0.81	30.52	23.41	23.71	1.45
	August	17.43	0.76	32.63	24.49	10.83	1.37

(1) $$y=f_{1}(X_{1})+f_{2}(X_{2})+\ldots +f_{m}(X_{m})+\epsilon \;$$

(2) $$f_{j}(X_{j})=\beta_{j0}+\beta_{j1}X_{j}+\beta_{j2}X_{j}^{2}+\ldots +\beta_{j\text{L}}X_{j}^{L}$$

In (Equation 1), the error term (ϵ) is assumed to follow a normal distribution with a mean of 0 and a standard deviation of $\sum_{j=1}^{m}\sigma_{j}^{2}$. Each independent variable in the PR model can be expressed using a polynomial function, as shown in (Equation 2), thereby enabling the representation of nonlinear effects. $\beta_{jl}$ denotes the coefficient associated with $X_{j}^{L}$, where L designates the maximum order of $f_{j}(X_{j})$. In this study, external weather variables, including solar radiation ( ${\text{X}}_{1}$), relative humidity ( ${\text{X}}_{2}$), daily maximum temperature ( ${\text{X}}_{3}$), daily minimum temperature ( ${\text{X}}_{4}$), precipitation ( ${\text{X}}_{5}$), and wind speed ( ${\text{X}}_{6}$), are selected as candidate independent variables. Five-fold cross validation is conducted using 175 days of data collected over two years to construct PR models under both heat and control regimes.

### 2.3 Simulation of tomato growth under heat treatment conditions

The APEX (Agricultural Policy/Environmental eXtender, APEX v.1501, Texas, USA) model was used for the crop growth model development. The APEX model is well-suited for simulating plant growth and soil water balance, as it represents processes such as light interception, biomass accumulation, biomass allocation to fruit, water use, and nutrient uptake. Moreover, it can simulate multiple constraints to growth, including water, temperature, and nutrient stress. The APEX is a daily process-based model that simulates hydrology and water quality in small to medium watersheds (Williams and Izaurralde, 2005). Accordingly, the APEX model has been adapted to simulate crop growth and soil water balance in agricultural fields, including small experimental plots.

As data input, the APEX model requires information on soil, weather, cropping schedule, and crop parameters. The greenhouse weather variables derived from the climate model described in Section 2.2 were applied to the model. Total precipitation was set to 0. The average wind speed in the greenhouse was maintained at 1 ms^-1^ throughout the simulation years. The plants were cultivated in soil-based greenhouses. The soil type is ‘Weongog’. This soil is moderately thick, brown to dark brown loam to sandy loam. Detailed soil properties are available at the Korean Soil Information System (soil.rda.go.kr). Cropping management techniques, such as planting date, harvest date, and planting density, followed the experimental schedules outlined in Section 2.1.

Based on field measurement results, a total of 4 crop parameter sets for HR17 and HR24 under control and heat stress conditions were established. **Table 2** presents selected key crop parameters used in the simulations. WA represents the biomass-energy ratio, which predominantly affects yield increases per unit of intercepted photosynthetically active radiation, and is strongly linked to radiation use efficiency. Measurements indicated that the net photosynthesis rates were higher for plants under control conditions than those exposed to heat stress. Additionally, HR24 showed a higher net photosynthesis rate than HR17, which was factored into the determination of WA values for each parameter set. The WA values were calibrated during model fine-tuning to minimize error between measured and simulated yield over. Further details are provided in the results section. The harvest index was adjusted for each species and temperature regime. HR23 is recognized as a high heat-tolerant crop, so its optimal temperature was set to 30°C, while the other species was set at 28°C. The base temperature for all varieties was maintained at 10°C. DMLA values were selected based on observations and subsequently refined during model calibration. DLAP1 and DLAP2 were both derived from experimental measurements. DLAP1 and DLAP2 are the first and second points on optimal leaf area development curve, respectively. As outlined in Section 2.1, multiple leaf area measurements were conducted throughout the experimental period, with the resulting growth curve utilized to establish DLAP1 and DLAP2 values. RLAD, the leaf area index decline rate, and RBMD, the biomass decline rate, were each set at 0.1, indicating that the plants remained green at harvest. PHU, potential heat units, was assigned a value of 2000 for all varieties under both greenhouse conditions. An automatic irrigation system was implemented during the simulation to eliminate water stress.

**Table 2:** Some important crop parameters of two tomato accessions, HR17 and HR24, under control and heat stress conditions used in the APEX model.

Parameter	Description	HR17-con	HR17-heat	HR24-cont	HR24-heat
WA	Biomass-Energy ratio: the maximum potential growth rate per unit of intercepted photosynthetically active radiation	48	40	50	45
HI	Harvest index	0.8	0.62	0.7	0.4
TB	Optimum temperature for plant growth(°C)	28	28	30	30
TG	Lowest temperature for plant growth(°C)	10	10	10	10
DMLA	Maximum potential leaf area index	7	3.82	12	7
DLAP1	First point on optimal leaf area growth curve	15.05	15.05	15.05	15.05
DLAP2	Second point on optimal leaf area growth curve	45.38	45.35	45.41	45.29
RLAD	Leaf area index reduction rate parameter	0.1	0.1	0.1	0.1
RBMD	Biomass-to-energy ratio reduction rate parameter	0.1	0.1	0.1	0.1

The measured total fruit yields were compared with the simulated yields for model calibration and validation. The total fruit yields were calculated as the sum of the total dry fruit yields from the 35^th^, 55^th^, and 77^th^ days of heat treatment. The fruit yield was determined by multiplying the dry fruit weight per plant by the plant density of 4.7 plants per m^2^. Additionally, leaf area development for both varieties under both treatment conditions during the experimental periods was analyzed to assess model accuracy. Due to the limited number of yield data points, yield data from all varieties and treatment conditions were combined to calculate the RMSE (root mean square errors), R^2^, and PBIAS(percent bias)value. And percent bias was calculated by subtracting observed yield from simulated yield and dividing by observed yield.

### 2.4 Climate change scenarios simulation

This study analyzed the impacts of climate change on greenhouse temperatures and yield production. The KACE climate change model, version KACE-1-0-G, was applied to project future climate conditions at the study site. The KACE model was developed by the National Institute of Meteorological Sciences/Korea Meteorological Administration (NIMS National Institute of Meteorological Sciences and Korea Meteorological Administration, 2021). The K-ACE can simulate the Earth’s climate system, including atmospheric and oceanic circulation patterns, and is extensively used for studying greenhouse gas impacts. Two scenarios were evaluated: SSP245 and SSP585. The SSP245 scenario reflects a “middle of the road” pathway, indicating moderate challenges regarding mitigation and adaptation. The SSP585 scenario describes an “Inequality-A Road Divided” pathway, reflecting a low mitigation challenge (Peng and Li, 2021) and high adaptation challenges. The CO_2_ concentrations for SSP245 and SSP585 are projected to reach 567 ppm and 1089 ppm, respectively.

The yields from 2022 to 2023 were compared with projected yields under SSP245 and SSP585 scenarios from 2030 to 2039 (10 years). Since it is assumed that water availability will not increase in the future, the irrigation amount for each variety in the climate change simulation was maintained at the maximum level used in 2022-2023. For HR17, in simulation setting, the maximum irrigation amount can be reached to 4000 mm annually, while HR24 can be received 6000 mm. PHU values were determined based on the accumulated radiation during growth periods, enabling continuous plant growth under climate change conditions. PHU values for SSP245 and SSP585 scenarios were set at 2600 and 2800, respectively. Since CO_2_ concentration can be regulated in the greenhouse, it was maintained at 360 ppm in both scenarios.

## 3 Results

### 3.1 Morphological and yield measurements

Under heat stress conditions, both tomato accessions, HR17 and HR24, experienced significant yield reductions (**Table 3**). The total fresh weight and fresh fruit yields of both accessions were significantly decreased under heat stress (*P* < 0.05). Fresh weights and fresh fruit yields of HR17 were reduced by 24 and 28% in heat stress condition, respectively. Fresh weights and fresh fruit yields of HR24 were reduced by 26 and 45% in heat stress condition, respectively. However, the total fresh weights of HR17 and HR24 differed significantly from each other, whereas there was no significant difference in fresh fruit yield between the accessions. Two accession had different growth patterns due to genotypic variation. HR24 accumulated more biomass than HR17 in both control and heat treatments. This indicates that HR24 allocated a greater proportion of its biomass to non-fruit components, resulting in a lower harvest index for HR24. Measurement of plant height and stem thickness showed that HR24 was taller and had thicker stems than HR17 under both heat and control conditions (*P* = 0.035 for height and *P* = 0.049 for stem thickness). HR24 also exhibited nearly twice the leaf area of HR17 (*P* = 0.0038). Heat treatment did not significantly influence the leaf area index, height, and stem thickness. **Supplementary Figure 1** shows the changes in plant heights and stem thickness of HR17 and HR24 across experimental days under control and heat stress conditions. Under heat stress conditions, HR17 continuously increased, while HR17 reached its maximum height at 40^th^ days. Stem thickness of HR17 under heat treatment was higher than control after 40^th^ days of treatment, while stem thickness of HR24 under heat stress was lower than control after 50^th^ days of treatment. Moisture content was greater in HR17(91 ± 0.77 in control and 90 ± 0.11 in heat) than in HR24 (89± 1.57 in control and 87 ± 0.63 in heat)(*P* = 0.0038). Additionally, the moisture content of both accessions was slightly reduced under heat treatment conditions. In summary, HR24 produced more overall biomass but allocated less to fruit yield, while HR17 allocated 60% of its total fresh weight to fruit production. HR24 recorded higher fresh weight than HR17, leading to greater values in biomass-related traits such as leaf area, height, and stem thickness.

**Table 3:** Yield components and morphological characteristics of two tomato accessions, including HR17 and HR24, on 77th days of heat treatment in control and heat greenhouses.

Treatment	Genetic accessions	Total fresh weight (kg/plant)	Fresh fruit yield (kg/plant)	Moisture content (%)	Leaf area index	Harvest index	Height (m)	Stem thickness (mm)
Control	HR17	2.5	1.63	91	3.24	0.65	1.9	15.8
	HR24	3.16	1.5	89	5.93	0.48	2.33	19.2
Heat	HR17	1.89	1.17	90	2.77	0.62	2.12	16.3
	HR24	2.34	0.83	87	4.8	0.35	2.27	17.2
ANOVA	Heat treatment(T)	0.0094	0.0177	0.038	n.s.	n.s.	n.s.	n.s.
	Accessions (A)	0.026	n.s.	0.0038	0.0038	0.0042	0.035	0.049
	T x A	n.s.	n.s.	n.s.	n.s.	n.s.	n.s.	n.s.

A summary of the analysis of variance (ANOVA) for the variables of two accessions was listed. The term “n.s.” indicates insignificant difference at alpha=0.05.

**Figure 2:**
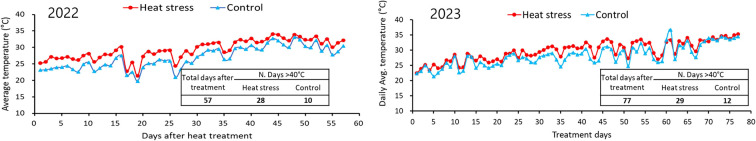
Effects of 0–15 days of heat treatments on net photosynthesis rate (A) of two tomato accessions, HR17 and HR24, grown under heat and control conditions.

Net photosynthesis rates (A) of two tomato accessions, HR17 and HR24, were evaluated (**Figure 2**). Both accessions exhibited reduced A values under heat treatment, indicating that photosynthetic efficiency declined upon exposure to heat stress. The HR17 accession maintained net photosynthesis rates around 20 µmol m^-2^s^-1^ during heat treatment, while HR24 showed a decline in photosynthesis rates from 20 to 18 µmol m^-2^s^-1^ after 13^th^ days of heat treatment.

### 3.2 Development of greenhouse climate model

Overall, greenhouse temperatures exceeded those recorded outside the greenhouses (**Table 4**). In 2022, the temperatures inside greenhouses were 1-2°C higher than in 2023; this difference corresponds to a nearly fourfold increase in total rainfall in 2023 (1269 mm), although specific rainfall data are not shown. In the heat treatment greenhouse, the average maximum temperature was 40.65°C in 2022 and 38.63°C in 2023, while the control greenhouse-maintained temperatures near 36-37°C in both years. The highest temperatures inside greenhouses were 8-10°C greater than those recorded outside. Minimum temperatures inside greenhouses were also marginally higher than ex-ternal temperatures. However, unlike temperature, humidity levels in greenhouses were 2-5% lower than outside. These elevated temperatures may contribute to increased dryness within greenhouses.

**Table 4:** Average maximum and minimum temperatures(°C) and humidity (%) between May and August 2022–2023 in control and heat treatment greenhouses and outside greenhouses.

Location	Greenhouse				Outside	
Treatment	Control		Heat treatment			
Year	2022	2023	2022	2023	2022	2023
Maximum temperature(°C)	37.36 ± 4.71	36.56 ± 5.45	40.65 ± 3.39	38.63 ± 4.47	29.83 ± 3.21	29.67 ± 3.42
Minimum temperature(°C)	21.5 ± 4.35	21.62 ± 3.65	22.2 ± 4.23	22.35 ± 3.67	21.28 ± 4.04	21.27 ± 3.42
Humidity (%)	68 ± 8.31	73 ± 9.96	68 ± 7.84	71.94 ± 9.07	73 ± 10.8	75 ± 9.6

Utilizing outside weather data (see **Table 1**) as an independent variable, PR modeling was conducted to describe the nonlinear relationships with the dependent variables identified in **Table 4**. (Equation 3) presents the PR model for estimating greenhouse relative humidity (RH_control) under control conditions, while (Equation 4) provides the PR model for greenhouse relative humidity estimation (RH_heat) under heat conditions. Other climate parameters, including daily maximum temperature, daily minimum temperature, precipitation, and wind speed, are excluded from both PR models because their Pearson correlation coefficients with RH_heat and RH_control are not statistically significant at a 0.05 confidence level. According to the regression coefficients table for both control and heat-treated conditions (**Supplementary Tables S1, S2**), only X_1_, X_2_, and X_5_ are selected as independent variables that are significant for changes in greenhouse relative humidity at the 0.05 significance level. Considering that precipitation (X_5_) has the Pearson correlation coefficient of - 0.70 (very strong correlation) with solar radiation (X_1_) in both conditions, it is appropriate to select X_1_ and X_2_ as independent variables to develop the PR models. The developed PR models using the two variables finally selected are as shown in (Equations 3, 4).

(3) $$RH_{control}=0.539429-0.009796{\text{X}}_{1}+0.000204{\text{X}}_{1}^{2}+0.475429{\text{X}}_{2}$$

(4) $$RH_{heat}=0.698088-0.012741{\text{X}}_{1}+0.000199{\text{X}}_{1}^{2}+0.277417{\text{X}}_{2}$$

The R^2^ value (coefficient of determination) for (Equation 3) is 0.90, and for (Equation 4) it is 0.89, demonstrating that both models provide strong predictive performance, adequately accounting for data variability.

The PR models for estimating greenhouse maximum temperature under the two conditions (Tmax_control and Tmax_heat) are developed as shown in (Equations 5, 6), and the R^2^ values were 0.84 and 0.78, respectively. Only solar radiation ( ${\text{X}}_{1}$), daily maximum temperature ( ${\text{X}}_{3}$), and daily minimum temperature ( ${\text{X}}_{4}$), which are suitable for the significance level of 0.05, are selected as independent variables and included in the PR models (**Supplementary Tables S3, S4**). Although precipitation (X_5_) is significant at the 0.05 significance level, it is excluded because it has the Pearson correlation coefficient of - 0.54 (strong correlation) with solar radiation. The PR models based on the selected three variables are as shown in (Equations 5, 6).

(5) $$Tmax_{control}=1.78009+0.80236{\text{X}}_{1}-0.01807{\text{X}}_{1}^{2}+0.79777{\text{X}}_{3}+0.00825{\text{X}}_{4}^{2}$$

(6) $$Tmax_{heat}=13.19313+0.82980{\text{X}}_{1}-0.01929{\text{X}}_{1}^{2}+0.54994{\text{X}}_{3}+0.00511{\text{X}}_{4}^{2}$$

The greenhouse minimum temperature estimation models are developed as shown in (Equations 7, 8), and the R^2^ values are 0.98 and 0.97, respectively. Unlike the greenhouse humidity and greenhouse maximum temperature models, it has a multivariate linear regression model with a linear relationship, and only solar radiation ( ${\text{X}}_{1}$), relative humidity ( ${\text{X}}_{2}$), daily maximum temperature ( ${\text{X}}_{3}$), daily minimum temperature ( ${\text{X}}_{4}$), and wind speed ( ${\text{X}}_{6}$) are included in the models as independent variables at a significance level of 0.05 (**Supplementary Tables S5, S6**). Notice that (Equations 7, 8) represent the final models based on the five selected variables.

(7) $$Tmin_{control}=-4.8907-0.0277{\text{X}}_{1}+5.6565{\text{X}}_{2}+0.0939{\text{X}}_{3}+0.9181{\text{X}}_{4}+0.3364{\text{X}}_{6}$$

(8) $$Tmin_{heat}=-4.0764-0.0339{\text{X}}_{1}+6.2056{\text{X}}_{2}+0.1138{\text{X}}_{3}+0.8767{\text{X}}_{4}+0.2621{\text{X}}_{6}$$

### 3.3 Tomato growth model development and model validation

**Figure 3:**
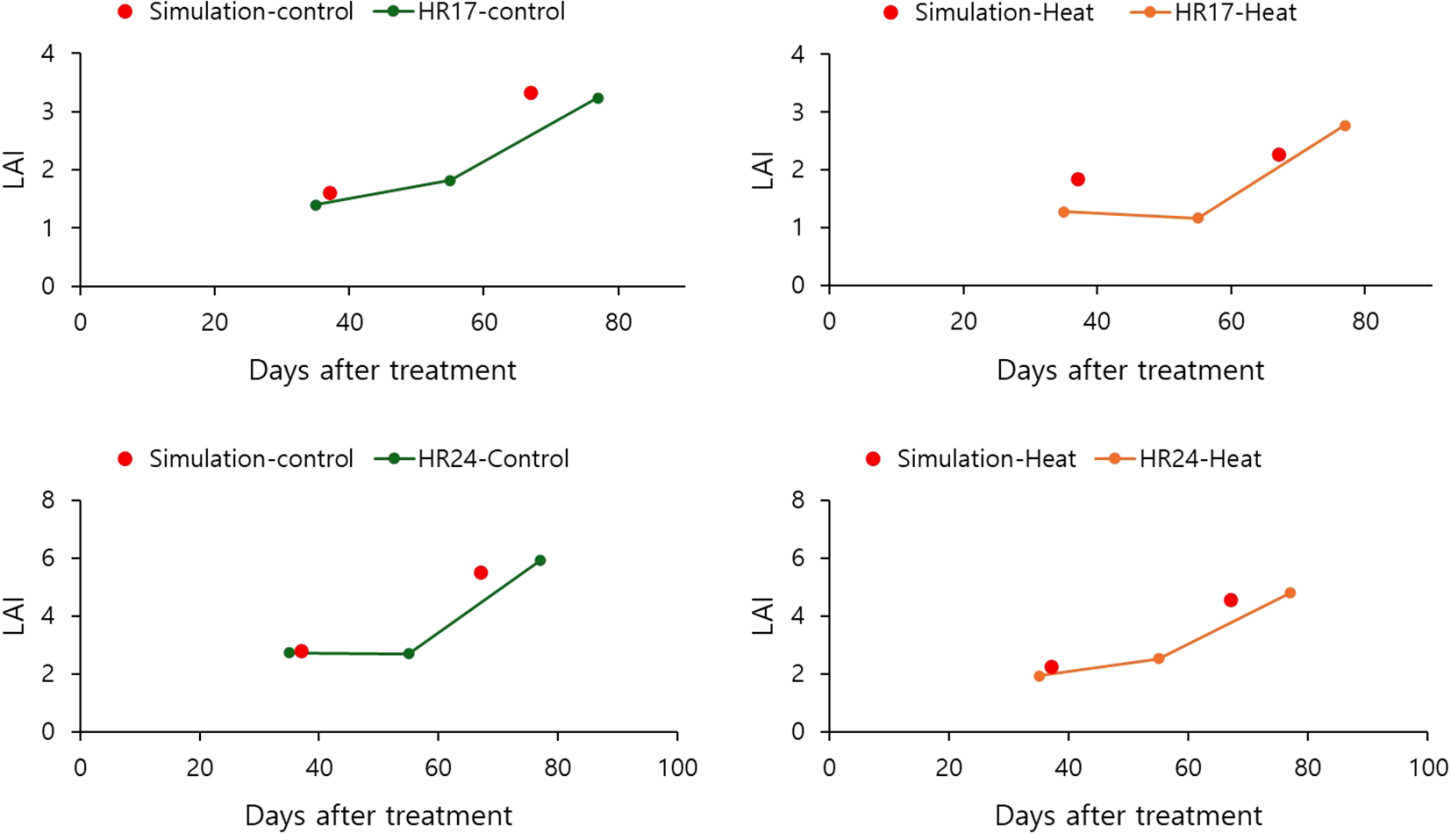
Comparison between simulated and measured LAIs of HR17 and HR24 in control and heat treatment greenhouses. The red circle denotes simulated LAI, and the line represents measured LAI on the 35^th^, 55^th^, and 77^th^ days of heat treatment.

Plant growth trajectories for HR17 and HR24 were analyzed using observed data to improve model accuracy. Leaf area measurements were recorded throughout the experimental period. The measured LAIs were utilized to calculate the crop parameters DLAP1 and DLAP2 (**Table 2**). Simulation results indicated that the modeled leaf area indices were closely aligned with the measured LAIs (**Figure 3**). In general, simulated LAIs were slightly higher than the measured values.

The tomato plant growth model was effectively used to assess the effects of heat stress on fruit yields (**Table 5**). The percentage bias remained below 3% across all simulations. The observed total fruit dry yields for HR17 and HR24 grown in the control greenhouse were 9.09 and 8.24 Mg ha^-1^, respectively, while the corresponding simulated yields were 8.88 and 8.00 Mg ha^-1^. Under heat stress, measured yields for HR17 and HR24 were 5.94 and 5 Mg ha^-1^, respectively, compared to simulated yields of 5.9 and 4.95 Mg ha^-1^.

**Table 5:** Comparison of measured and simulated dry fruit yields of HR17 and HR24 under control and heat treatment conditions in greenhouses during 2022-2023.

Treatment	Accession	Observed fruit dry yield	Predicted fruit dry yield	Percent bias (%)
		Mg ha^-1^	Mg ha^-1^	
Control	17	9.09	8.88	-2.34
	24	8.24	8.00	-2.93
Heat	17	5.94	5.90	-0.70
	24	5.00	4.95	-1.00
			RMSE	0.03 Mg/ha
			R^2^	0.99

Percentage bias, root mean square (RMSE), and R^2^ are reported.

### 3.4 Tomato yields under projected climate change scenarios

Following the successful establishment of the tomato growth model, the impacts of heat stress under climate change scenarios SSP245 and SSP585 were analyzed. **Table 6** presents the projected changes in temperatures and evapotranspiration. In the historical period, maximum temperatures peaked at 40°C in July, whereas in both climate change scenarios, maximum temperatures exceeded 40°C in June. Elevated temperatures led to increased evapotranspiration rates in climate change scenarios compared to the historical period. For instance, in July, the historical evapotranspiration rate was 190 mm, rising to 215 mm for SSP245 and 202 mm for SSP585. Furthermore, temperature declines typically observed after July in the historical period are replaced by a continuous temperature increase after July in the climate change scenarios.

**Table 6:** Monthly average greenhouse maximum and minimum temperatures, and evapotranspiration during the May–August tomato growing season in history (2022-2023), SSP245 (2030-2039), and SSP585 (2030-2039) scenarios.

Scenarios	Month	5	6	7	8
Historical period (2022-2023)	Maximum temperature (°C)	29.58	38.68	40.45	35.18
	Minimum temperature (°C)	13.88	20.56	24.92	24.19
	Evapotranspiration (mm)	26.58	73.08	190.07	105.72
SSP245 (2030-2039)	Maximum temperature (°C)	37.1	40.1	42.03	42.61
	Minimum temperature (°C)	14.36	20.87	25.44	26.02
	Evapotranspiration (mm)	43.9	122.06	214.89	7.55
SSP585(2030-2039)	Maximum temperature (°C)	36.59	40.07	41.37	42.34
	Minimum temperature (°C)	14.51	20.78	25.07	25.6
	Evapotranspiration (mm)	40.81	113	201.82	30.17

Simulation results indicated that HR24 exhibited greater tolerance to future climate conditions. Dry fruit yields of HR24 increased under the SSP245 and SSP585 scenarios, while HR17 yields declined under projected climate conditions (**Table 7**). Irrigation amount needs for both accessions will increase in future climate scenarios to sustain fruit yields comparable to those in historical periods, resulting in water use efficiency decreases in both climate change scenarios (**Table 7**). Projected fruit yields for both accessions were similar between SSP245 and SSP585 scenarios, possibly due to the cultivation of plants in environmentally controlled conditions.

**Table 7:** Simulated average dry fruit yields and water use efficiency for historical (2022-2023) and climate change scenarios SSP245 (2030-2039) and SSP585 (2030-2039).

Treatment	Accession	Simulated dry fruit yield			Water use efficiency		
		Historical yield	SSP245	SSP585	Historical yield	SSP245	SSP585
		Mg/ha	Mg/ha	Mg/ha	kg/mm	kg/mm	kg/mm
Thermal energy	17	5.94	4.68	4.69	14.73	11.06	11.14
	24	5.00	6.49	6.27	10.41	10.25	10.08

## 4 Discussion

This study is the initial effort to simulate the effects of prolonged heat stress on tomato production in South Korea. A process-based model was adopted to achieve this objective due to its demonstrated accuracy in evaluating the impacts of various environmental stresses, such as temperature and water stress, on crop yields. The equations in the process-based model were based on experimental datasets or were calibrated using observed data, which may allow the model to produce more realistic projections under heat stress scenarios (Lobell and Asseng, 2017). Numerous simulation studies have also employed process-based models to assess temperature effects on the yields of major crops (Rosenzweig et al., 2014; Moore et al., 2017). Nevertheless, the majority of these simulations have focused on field crops. Only a few studies have developed process-based models specifically for greenhouse systems (Katzin et al., 2022; Park et al., 2025). Since greenhouses are climate-controlled environments with characteristics that differ substantially from open-field conditions, accurate greenhouse weather data is crucial for enhancing the precision of greenhouse system simulations. In this re-search, a greenhouse climate model was successfully developed, and the weather data produced were incorporated into the plant growth model.

Plant parameters and growth responses were monitored to improve the model’s accuracy as the plants experienced heat stress for nearly two months. The two tomato accessions, HR17 and HR24, displayed distinct growth dynamics. HR17 was inclined to allocate more resources toward fruit production than overall biomass, whereas HR24 accumulated more biomass relative to fruit output. According to the parameterization, the harvest index values for HR17 and HR24 were approximately 0.6 and 0.4, respectively. Because HR24 produced greater biomass than HR17, this resulted in higher measurements for plant height, stem thickness, and leaf area compared to HR17. Additionally, HR24 exhibited a faster growth rate compared to HR17. The DLAP2 value observed for HR24 was 45.41, while for HR17 it was 45.38 under control greenhouse conditions. These findings indicate that HR24 and HR17 could achieve 41% and 38% of their total yield production at 45% of the growing season. Under heat stress conditions, the productivity of both accessions declined. At the 45% point of the growing season, heat stress had the most pronounced effect on HR24, with a DLAP2 of 45.29 under these conditions. This suggests that HR24 experienced inhibited growth during the first month of heat exposure.

However, following the initial month of heat exposure, HR24 exhibited accelerated growth, evidenced by a sharp increase in its leaf area index between the 55th and 77th days of heat treatment. By the 77th day of heat treatment, HR24 had only reduced its fresh weight by 25% under heat stress conditions. The sustained growth observed under prolonged heat stress may be attributed to priming, in which previous heat exposure alters the response to subsequent stress events (Bruce et al., 2007; Conrath, 2011; Vriet et al., 2015; Avramova, 2015; Hilker et al., 2016). Zhou et al. (2020) reported that priming in tomato plants enhances their ability to cope with heat stress by increasing evaporation rates and reducing leaf temperatures. Heat-response genes (HRGs) can play a critical role in plant adaptation mechanisms. Under heat stress, expression of Heat shock factors-related genes in tomato increased rapidly (Mishra et al., 2002). Fragkostefanakis et al. (2016) found that HSFA2 plays a crucial role in the priming mechanism of tomato plants, which maintains the pollen viability throughout the process of microsporogenesis. Both accessions showed a decrease in photosynthetic capacity under heat stress. Consequently, within the crop parameter set, the values of WA, biomass-energy ratio, for heat-stressed plants were lower than those from the control group. As HR24 accumulated more biomass than HR17, its net photosynthesis rates remained slightly higher. This relationship explains why WA in HR17 was lower than WA in HR24. This means that HR24 produced more biomass than HR17 with same amount of intercepted photosynthetically active radiation.

The growth model effectively simulated accessions under control and heat stress conditions in the greenhouse. The modeled growth trajectories of HR17 and HR24 also closely matched their observed leaf area values in both greenhouse environments. Under future climate scenarios, with prolonged high temperatures (above 40°C) extending beyond July, HR17 experienced significant yield reductions. In contrast, the HR24 accession adapted well to elevated temperatures by producing higher yields than those in historical periods. These results suggest that plants with a higher proportion of accumulated biomass may display increased tolerance to heat stress. HR24 produced more leaves to increase leaf area index, resulting in higher photosynthesis efficiency than HR17 for a long-term period. The greater elongation of stem and leaves can provide physiological benefits to avoid the heat dissipation (Gray et al., 1998). Moreover, higher photosynthesis efficiency can sustain gas exchange rate under heat stress (Craita and Tom, 2013). Nevertheless, both accessions experienced decreased water use efficiency under heat stress.

Although the developed model can evaluate the effects of prolonged heat stress on tomato growth and production, several limitations were encountered during its development. It is widely recognized that fruit quality is strongly influenced by temperature. Mesa et al. (2022) found that elevated temperatures can enhance fruit quality through increased total soluble solids and vitamin C levels in fruits. Nevertheless, Lokesha et al. (2019) presented opposing findings, indicating that heat stress diminished total carotenoid and lycopene contents in tomato fruits. Furthermore, their study highlighted that heat stress’s impact on fruit quality depended on plant genotype. Only few modeling studies have been conducted to simulate greenhouse tomato yields. Fang et al. (2022) used mathematical models to simulate greenhouse tomato growth curve using growing degree days. In the current model, tomato fruit yields and other heat tolerant related parameters were included in the simulations. Future research can focus on formulating an empirical model that quantifies the relationship between fruit quality and temperature based on experimental data, which can then be incorporated into the tomato model developed in this study. Additionally, although hundreds of different tomato varieties are commercially available, this study developed models for only two accessions. Further investigations into cellular or molecular levels (e.g. leaf stomatal density, antioxidant enzyme activity, heat shock protein expression) should encompass additional accessions with varying growth habits to improve the model’s applicability.

## 5 Conclusions

A plant growth model for two tomato accessions, HR17 and HR24, was developed in this study. These two accessions had different growth patterns under heat stress conditions. HR24 tended to have more biomass over fruits, while HR17 accession had higher fruits than biomass. The model aims to simulate tomato production in greenhouse environments under different climate change scenarios, which will help farmers to select better accession in rising temperature condition. Model development was grounded in experimental data collected from greenhouse trials. The resulting model was able to differentiate the growth responses of HR17 and HR24 under heat stress conditions. HR24 exhibited a larger plant stature than HR17 and demonstrated a greater proportion of biomass allocated to fruit yields. In the parameter set, HR24 was characterized by a lower HI. In contrast, HR17 attained a harvest index above 0.6, resulting in fruit production nearly equivalent to HR24. The model can realistically simulate the fruit yields of HR17 and HR24 with less than 3% percent bias. Under projected future climate conditions, HR24 is anticipated to show greater heat stress tolerance due to its higher yields under increased temperatures. This performance may be attributed to its larger biomass production, such as increased leaf area, stem thickness, and height, which help maintain photosynthetic capacity and increase resilience to thermal stress. These results can play critical roles in development of heat tolerant breeding programs. Currently, few models are available to simulate greenhouse systems. This study contributes significantly to advancing greenhouse system simulation. Moreover, as most horticultural crops in South Korea are grown in greenhouses due to constrained agricultural land, the developed simulation will provide valuable support to farmers by enabling more accurate yield forecasting, im-proved operational decision-making (e.g., optimizing planting or harvest dates), and refined technical management (e.g., temperature regulation).

## Data Availability

The original contributions presented in the study are included in the article/**Supplementary Material**. Further inquiries can be directed to the corresponding author.
