# Supplementary material for: Morphological analysis-based yield modeling in greenhouse grown cherry tomato (Solanum lycopersicum) under prolonged heat stress
Source: Front Plant Sci. 2025 Dec 19;16:1730694. doi: 10.3389/fpls.2025.1730694 (PMC12757247; doi:10.3389/fpls.2025.1730694)
Supplement: Supplementary file 1 [file DataSheet1.docx]

Supplementary Material

# Supplementary Data

Supplementary Material should be uploaded separately on submission. Please include any supplementary data, figures and/or tables.

Supplementary material is not typeset so please ensure that all information is clearly presented, the appropriate caption is included in the file and not in the manuscript, and that the style conforms to the rest of the article.

# Supplementary Figures and Tables

## Supplementary Figures

##
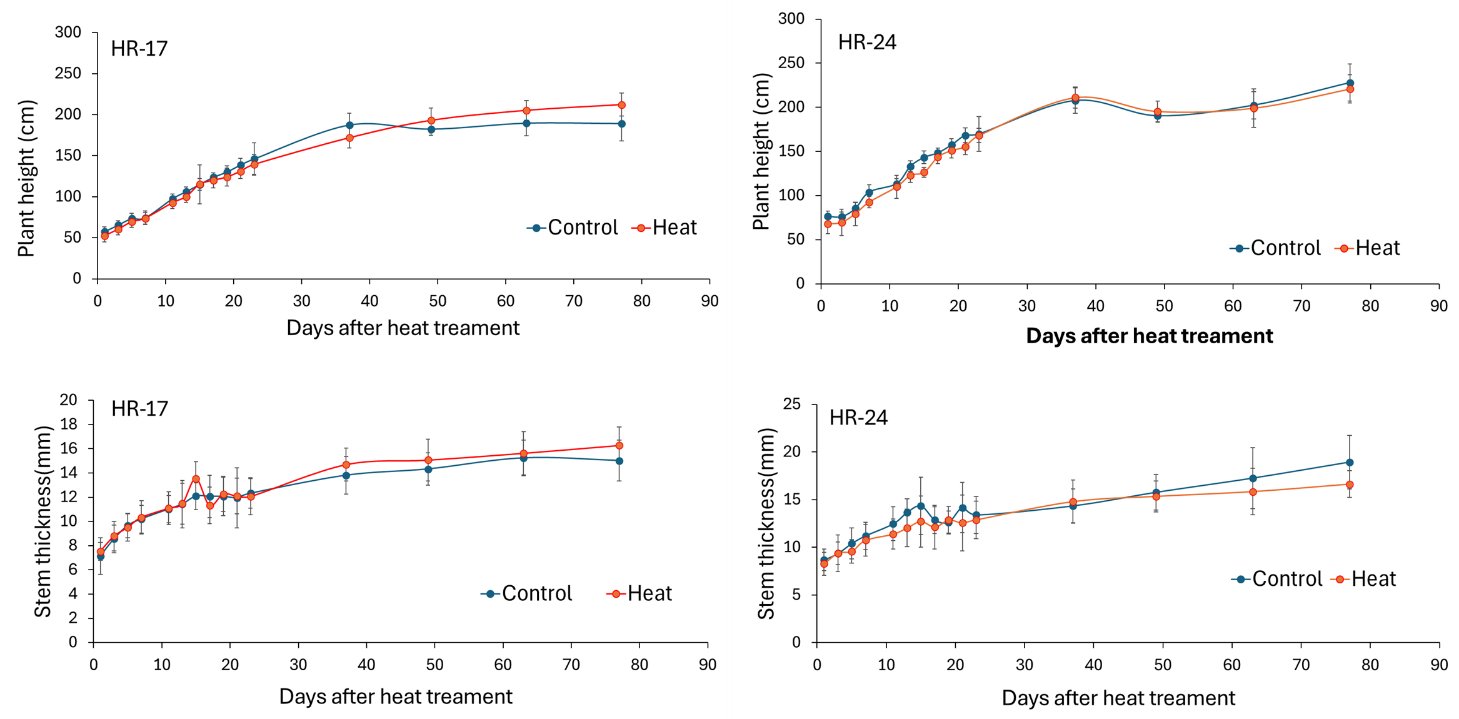


## Supplementary Figure 1 (SFigure1). Plant height and stem thickness of two cherry tomato accessions, HR-17 and HR-24, in control and heat stress conditions in 2022 and 2023.

## Supplementary Tables

- Supplementary Table 1 (Stable1). Linear regression coefficients table of greenhouse relative humidity in the control environment. The weather variables are solar radiation (X_1_), relative humidity (X_2_), daily maximum temperature (X_3_), daily minimum temperature (X_4_), precipitation (X_5_), and wind speed (X_6_).

| Category | Coefficients | Standard error | *t* statistic | *P*-value | Lower 95% | Upper 95% |
| --- | --- | --- | --- | --- | --- | --- |
| Intercept | 0.311683 | 0.044878 | 6.945193 | 7.91x10^-11^ | 0.223086 | 0.400279 |
| X_1_ | -0.00375 | 0.000633 | -5.92343 | 1.73 x10^-8^ | -0.005 | -0.0025 |
| X_2_ | 0.634949 | 0.039427 | 16.10433 | 6.85 x10^-36^ | 0.557113 | 0.712786 |
| X_3_ | 0.000768 | 0.001748 | 0.439092 | 0.66 | -0.00268 | 0.00422 |
| X_4_ | -0.00169 | 0.001567 | -1.0814 | 0.28 | -0.00479 | 0.001399 |
| X_5_ | 0.00047 | 0.000107 | 4.409195 | 1.85 x10^-5^ | 0.00026 | 0.00068 |
| X_6_ | -0.00065 | 0.00624 | -0.10443 | 0.92 | -0.01297 | 0.011667 |

- Supplementary Table 2 (Stable2). Linear regression coefficients table of greenhouse relative humidity in the heat environment. The weather variables are solar radiation (X_1_), relative humidity (X_2_), daily maximum temperature (X_3_), daily minimum temperature (X_4_), precipitation (X_5_), and wind speed (X_6_).

| Category | Coefficients | Standard error | *t* statistic | *P*-value | Lower 95% | Upper 95% |
| --- | --- | --- | --- | --- | --- | --- |
| Intercept | 58.73868 | 4.300647 | 13.6581 | 4.78 x10^-29^ | 50.24841 | 67.22896 |
| X_1_ | -0.63395 | 0.060683 | -10.447 | 5.33 x10^-20^ | -0.75375 | -0.51415 |
| X_2_ | 35.05912 | 3.778341 | 9.278972 | 8.40 x10^-17^ | 27.59998 | 42.51827 |
| X_3_ | -0.09577 | 0.167557 | -0.57156 | 0.57 | -0.42656 | 0.23502 |
| X_4_ | -0.00676 | 0.150176 | -0.045 | 0.96 | -0.30323 | 0.289718 |
| X_5_ | 0.038574 | 0.010213 | 3.776862 | 2.20 x10^-4^ | 0.018411 | 0.058736 |
| X_6_ | -0.55659 | 0.597969 | -0.9308 | 0.35 | -1.73709 | 0.623909 |

- Supplementary Table 3 (Stable 3). Linear regression coefficients table of greenhouse maximum temperature in the control environment. The weather variables are solar radiation (X_1_), relative humidity (X_2_), daily maximum temperature (X_3_), daily minimum temperature (X_4_), precipitation (X_5_), and wind speed (X_6_).

| Category | Coefficients | Standard error | *t* statistic | *P*-value | Lower 95% | Upper 95% |
| --- | --- | --- | --- | --- | --- | --- |
| Intercept | -4.80916 | 3.085666 | -1.55855 | 0.12 | -10.9008 | 1.282519 |
| X_1_ | 0.252291 | 0.043539 | 5.794577 | 3.3 x10^-8^ | 0.166337 | 0.338246 |
| X_2_ | 7.819202 | 2.710917 | 2.884338 | 4.44 x10^-3^ | 2.467349 | 13.17105 |
| X_3_ | 0.931436 | 0.12022 | 7.747746 | 8.45 x10^-13^ | 0.694099 | 1.168774 |
| X_4_ | 0.256314 | 0.10775 | 2.378782 | 0.02 | 0.043595 | 0.469032 |
| X_5_ | -0.01595 | 0.007328 | -2.17674 | 0.03 | -0.03042 | -0.00148 |
| X_6_ | -0.97087 | 0.429036 | -2.26292 | 0.02 | -1.81787 | -0.12388 |

- Supplementary Table 4 (Stable 4). Linear regression coefficients table of greenhouse maximum temperature in the heat environment. The weather variables are solar radiation (X_1_), relative humidity (X_2_), daily maximum temperature (X_3_), daily minimum temperature (X_4_), precipitation (X_5_), and wind speed (X_6_).

| Category | Coefficients | Standard error | *t* statistic | *P*-value | Lower 95% | Upper 95% |
| --- | --- | --- | --- | --- | --- | --- |
| Intercept | 11.69973 | 2.987757 | 3.915891 | 1.31 x10^-4^ | 5.801344 | 17.59812 |
| X_1_ | 0.173081 | 0.042158 | 4.105559 | 6.28 x10^-5^ | 0.089854 | 0.256308 |
| X_2_ | 1.698864 | 2.624899 | 0.647211 | 0.52 | -3.48317 | 6.880902 |
| X_3_ | 0.6561 | 0.116406 | 5.636324 | 7.20 x10^-8^ | 0.426294 | 0.885907 |
| X_4_ | 0.205085 | 0.104331 | 1.965719 | 0.05 | -0.00088 | 0.411054 |
| X_5_ | -0.02923 | 0.007095 | -4.11895 | 5.96 x10^-5^ | -0.04323 | -0.01522 |
| X_6_ | -0.02136 | 0.415423 | -0.05142 | 0.96 | -0.84148 | 0.798761 |

- Supplementary Table 5 (Stable 5). Linear regression coefficients table of greenhouse minimum temperature in the control environment. The weather variables are solar radiation (X_1_), relative humidity (X_2_), daily maximum temperature (X_3_), daily minimum temperature (X_4_), precipitation (X_5_), and wind speed (X_6_).

| Category | Coefficients | Standard error | *t* statistic | *P*-value | Lower 95% | Upper 95% |
| --- | --- | --- | --- | --- | --- | --- |
| Intercept | -4.97605 | 0.872705 | -5.70187 | 5.22 x10^-8^ | -6.69893 | -3.25317 |
| X_1_ | -0.03015 | 0.012314 | -2.44878 | 0.02 | -0.05446 | -0.00584 |
| X_2_ | 5.801436 | 0.766717 | 7.566595 | 2.40 x10^-12^ | 4.287795 | 7.315077 |
| X_3_ | 0.096567 | 0.034001 | 2.840086 | 5.07 x10^-3^ | 0.029442 | 0.163692 |
| X_4_ | 0.914651 | 0.030474 | 30.01371 | 2.10 x10^-69^ | 0.854489 | 0.974813 |
| X_5_ | -0.00151 | 0.002072 | -0.7281 | 0.47 | -0.0056 | 0.002583 |
| X_6_ | 0.356382 | 0.121342 | 2.936994 | 3.78 x10^-3^ | 0.11683 | 0.595934 |

- Supplementary Table 6 (Stable 6). Linear regression coefficients table of greenhouse minimum temperature in the heat environment. The weather variables are solar radiation (X_1_), relative humidity (X_2_), daily maximum temperature (X_3_), daily minimum temperature (X_4_), precipitation (X_5_), and wind speed (X_6_).

| Category | Coefficients | Standard error | *t* statistic | *P*-value | Lower 95% | Upper 95% |
| --- | --- | --- | --- | --- | --- | --- |
| Intercept | -4.19186 | 0.983738 | -4.26115 | 3.38 x10^-5^ | -6.13394 | -2.24978 |
| X_1_ | -0.0373 | 0.013881 | -2.68729 | 7.93 x10^-3^ | -0.0647 | -0.0099 |
| X_2_ | 6.401749 | 0.864265 | 7.407158 | 5.97 x10^-12^ | 4.695529 | 8.107969 |
| X_3_ | 0.117425 | 0.038327 | 3.063746 | 2.55 x10^-3^ | 0.04176 | 0.19309 |
| X_4_ | 0.871943 | 0.034352 | 25.38284 | 2.23 x10^-59^ | 0.804126 | 0.939759 |
| X_5_ | -0.00204 | 0.002336 | -0.87425 | 0.38 | -0.00665 | 0.00257 |
| X_6_ | 0.289077 | 0.136781 | 2.113435 | 0.04 | 0.019047 | 0.559107 |
